# Contractile actomyosin arcs promote the activation of primary mouse T cells in a ligand-dependent manner

**DOI:** 10.1371/journal.pone.0183174

**Published:** 2017-08-17

**Authors:** Jinsung Hong, Sricharan Murugesan, Eric Betzig, John A. Hammer

**Affiliations:** 1 Cell Biology and Physiology Center, National Heart, Lung and Blood Institute, National Institutes of Health, Bethesda, MD, United States of America; 2 Janelia Research Campus/HHMI, Ashburn, VA, United States of America; Semmelweis Egyetem, HUNGARY

## Abstract

Mechano-transduction is an emerging but still poorly understood component of T cell activation. Here we investigated the ligand-dependent contribution made by contractile actomyosin arcs populating the peripheral supramolecular activation cluster (pSMAC) region of the immunological synapse (IS) to T cell receptor (TCR) microcluster transport and proximal signaling in primary mouse T cells. Using super resolution microscopy, OT1-CD8^+^ mouse T cells, and two ovalbumin (OVA) peptides with different affinities for the TCR, we show that the generation of organized actomyosin arcs depends on ligand potency and the ability of myosin 2 to contract actin filaments. While weak ligands induce disorganized actomyosin arcs, strong ligands result in organized actomyosin arcs that correlate well with tension-sensitive CasL phosphorylation and the accumulation of ligands at the IS center. Blocking myosin 2 contractility greatly reduces the difference in the extent of Src and LAT phosphorylation observed between the strong and the weak ligand, arguing that myosin 2-dependent force generation within actin arcs contributes to ligand discrimination. Together, our data are consistent with the idea that actomyosin arcs in the pSMAC region of the IS promote a mechano-chemical feedback mechanism that amplifies the accumulation of critical signaling molecules at the IS.

## Introduction

T cells play a crucial role in adaptive immunity by recognizing pathogens and foreign entities that are presented by antigen presenting cells (APCs). This recognition process is initiated by the binding of the T cell receptor (TCR) on the T cell’s surface to an antigenic peptide presented by the major histocompatibility complex (pMHC) on the APC’s surface. This interaction results in the triggering of robust signaling cascades within the T cell downstream of TCR engagement that, together with signals generated by CD28 engagement and the interaction of the T cell’s integrin LFA-1 with ICAM-1 on the APC surface, drive robust T cell effector function. Remarkably, T cells are capable of sensing a very small number of antigenic-pMHCs in a sea of self-pMHCs, i.e. TCR-pMHC interaction is highly sensitive [1,2]. Moreover, the extent to which the T cell responds is altered dramatically by single amino acid residue differences in the short antigenic peptide, i.e., TCR-pMHC interaction is highly specific [3,4]. Efforts to model these two remarkable properties have led to the concept of kinetic proofreading [5], which has evolved to include versions that focus on enzymatic reactions (e.g. phosphorylation) [6,7], receptor conformational changes [8], and receptor dimerization/oligomerization [9,10]. In general, the concept of kinetic proofreading argues that T cell signaling is triggered by a series of reactions requiring continuous TCR–pMHC interaction such that these reactions are rapidly terminated whenever the interaction is lost. Direct support for these kinetic proofreading models is still lacking, however, and the models do not fully explain the mechanism underlying ligand discrimination.

One potentially critical component of kinetic proofreading that has recently drawn more attention is the role of force in regulating TCR–pMHC interaction [11–14]. An early clue to the idea that T cells sense force via TCR–pMHC interaction was the observation that T cells are readily activated by surface-anchored pMHC but not by soluble pMHC, implying that physical constraint is required for T cell activation [15,16]. This conclusion has gained strong support from follow-up studies employing a variety of biophysical techniques to test the contribution of force to TCR–pMHC interaction that include laser tweezers [17,18], hydrodynamic flow [19], polyacrylamide gels [20,21], micropipettes [22], atomic force microscopy [23], and biomembrane force probes [24,25]. The strong consensus from these studies is that the TCR senses the tension or stiffness resulting from its interaction with pMHC and then converts this force-readout into force-triggered intracellular calcium flux [18,19,22,24] and cytokine IL-2 production [17,20,21]. How this occurs at the molecular level has been directly addressed by biophysical studies showing that force prolongs the lifetime of TCR–pMHC interactions involving strong ligands by creating catch-bonds, and shortens the lifetime of TCR–pMHC interactions involving weak ligands by creating slip-bonds [17,24–26]. Inherent in this concept is the idea that force controls the rate of TCR dissociation from pMHC by testing the strength of TCR–pMHC interaction in a dynamic manner. In other words, force may act as a filter to gauge how well the TCR–pMHC bond resists applied tension. Less clear, however, is what cellular components generate and regulate this filtering force, and how this cellular force contributes to discriminating strong TCR–pMHC interactions from weak TCR–pMHC interactions.

A major cellular force that acts in the plane of the IS and likely contributes to TCR mechano-transduction and force-dependent ligand discrimination is the radially symmetric inward flow of actin observed in activated T cells using GFP-tagged actin as a dynamic reporter [27,28]. This robust centripetal actin flow, which occupies primarily the dSMAC portion of the IS, is generated by continuous, Arp2/3 complex-dependent branched nucleation at outer edge of the IS, and drives the formation of TCR microclusters (MCs) and their centripetal transport towards the cSMAC region of the IS [29–38]. Importantly, the assembly of branched actin networks has been shown to produce force both in vitro and in vivo [39]. Moreover, elegant biophysical studies have revealed that TCR MCs are frictionally coupled to the inward flow of actin at the IS [33,40], indicating that MCs indeed experience actin polymerization-dependent force (see also [41]).

A second major force acting in the plane of the IS that likely contributes to TCR mechano-transduction is that provided by the myosin 2-dependent contraction of actin filaments. Consistent with this idea, inhibiting the function of myosin 2 results in defects in IS formation, T cell: APC adhesion, calcium flux, early TCR signaling, and the force-dependent phosphorylation of lymphocyte-specific Crk-associated substrate (CasL) [42,43], although there is not complete consensus on this issue [31,44]. Absent from these earlier studies, however, was a clear understanding of where myosin 2 functions at the IS. Moreover, the nature of the actin network capable of propelling TCR MC movement across the pSMAC was a mystery because GFP-actin did not reveal obvious actin organization there [35]. Both of these ambiguities were resolved by Yi and colleagues, who identified concentric actin arcs decorated with myosin 2 in the pSMAC region of live Jurkat T cells using RFP-tagged myosin 2 and GFP-F-Tractin, an indirect reporter for F-actin, instead of GFP-actin [38]. Moreover, this second actin network was shown to be an endogenous structure, as it was readily apparent in untransfected T cells stained with phalloidin and antibodies to myosin 2. Importantly, the lamellipodial-like branched actin network comprising the dSMAC and the lamella-like actomyosin 2 arcs comprising the pSMAC were shown to exhibit distinct inward flow rates, and these rates were shown to match the distinct rates of centripetal TCR MC movement across these two regions in bilayer-engaged Jurkats T cells [38].

Most recently, Murugesan and colleagues [45] used several forms of super-resolution microscopy including total internal reflection structured illumination microscopy (TIRF-SIM) and 3D-SIM to show that the actin arcs in the pSMAC arise from linear actin filaments generated by one or more formins present at the distal edge of the IS (of note, this explains the poor labeling of arcs when GFP-actin is used [35], as formins do not incorporate modified actin monomers into filaments efficiently [46]). After traversing the outer dSMAC, these linear filaments were shown to be organized by myosin 2 bipolar filaments into concentric arcs that populate the pSMAC and possess the anti-parallel organization required for contraction. Importantly, quantitative, fixed-cell 3D-SIM showed that the open, active form of LFA-1 often aligns with arcs while TCR MCs commonly reside between arcs, and live-cell TIRF-SIM showed that TCR MCs are swept inward across the pSMAC by arcs. Consistent with these observations, disrupting actin arc formation via formin inhibition or actin arc organization via myosin 2 inhibition resulted in less centralized TCR MCs, miss-segregated integrin clusters, decreased T: B cell adhesion frequency, and diminished proximal TCR signaling. Finally, actomyosin arcs were shown to be a component of synapses formed by primary mouse T cells as well. Indeed, rather than being difficult to detect or absent, actomyosin arcs are an even more prominent component of the mouse CD8^+^ T cell IS than the Jurkat T cell IS, arguing that this contractile structure probably plays a significant role in promoting T cell effector function *in vivo*.

Here we sought to define the contribution made by these force-producing actomyosin arcs to ligand discrimination by the TCR in the context of primary T cells. Using super resolution imaging, we visualized the actomyosin networks that form in OT1 monoclonal mouse T cells in response to a strong and a weak ligand, and then correlated the observed differences to differences in the phosphorylation state of CasL, the extent of TCR–pMHC centralization, and the robustness of early TCR signaling and T cell-APC adhesion. Together, our data support the growing consensus [11,13,47] that actomyosin-dependent force generation plays an important role in force-regulated ligand discrimination at the IS.

## Materials and methods

### Cells and reagents

OT1 TCR transgenic and C57BL/6 (Jackson Laboratories, ME) mice were maintained in a Specific Pathogen Free facility and euthanized by cervical dislocation following prior narcotization with CO_2_. Animal husbandry and euthanasia were performed in accordance with protocols approved by the National Human Genome Research Institute Animal Use and Care Committee at the National Institutes of Health. OT1 T cells were obtained from 4-6-week-old OT1 mouse spleens and lymph nodes, as described previously [48]. The cells were stimulated with 10 nM OVA^257-264^ peptide (InnoPep, San Diego, CA) for three days in RPMI medium 1640 supplemented with 10% FBS, 50 mM beta-mercaptoethanol, 2 mM L-glutamine, 1 mM sodium pyruvate, and 50 U/mL penicillin and streptomycin (GIBCO, Grand Islands, NY), and then cultured for an additional for two days in media supplemented with 50 U/ml IL-2 to complete the activation process. Activated OT1 T cells were separated from dead cells using lymphocyte separation medium (Lonza, Walkersville, MD) before use. The cell line EL4 was used as the APC. For pMHCs, we obtained synthesized recombinant H-2K^b^ pMHC monomers from the National Institutes of Health Tetramer Core Facility at Emory University that contained either the chicken ovalbumin peptide^257-264^ SIINFEKL (OVA) or the mutant version SIIGFEKL (G4). SIINFEKL and SIIGFEKL peptides were synthesized by InnoPep (San Diego, CA). The plasmids for expression of the dynamic F-actin reporter GFP-F-Tractin and a Halo-tagged version of the myosin 2 heavy chain were described previously [38,45,49]. The Halo dye 567 was a gift of Luke Lavis (Janelia Research Campus/HHMI, Ashburn, VA).

### Surface preparation

To activate OT1 T cells with pMHC monomers, 8-well coverglass chamber slides (Nunc LabTek II Chambered #1.5, Thermo Scientific, Waltham, MA) were incubated sequentially for one hour each with 1 mg/ml BSA-biotin, 1 mg/ml streptavidin (SA), and 10 μg/ml pMHCs plus 5 μg/ml mouse recombinant CD80 (Sino Biological, Beijing, China). Three wash steps were performed between each addition. Activation of OT1 T cells on glass-supported planar lipid-bilayer was performed as described previously [45,50]. In brief, biotin-CAP-PE and DGS-Ni were passed through a mini-extruder kit (Avanti Polar Lipids, Alabaster, AL) to generate liposomes, which were then mixed and added to a flow chamber formed between a clean glass slide and a cover slip. Following a one hour incubation at RT, the chamber was blocked using 5% w/v casein in 1X PBS. Finally, the flow chamber was incubated with 1 mg/ml SA, 10 μg/ml pMHCs, and 10 μg/ml recombinant mouse His-ICAM-1.

### Super-resolution imaging

To fix and stain OT1 T cells during IS maturation, cells activated on either coverglass chamber slides or glass-supported lipid-bilayers for 7 min were fixed using 4% formaldehyde in 1X PBS, permeabilized using 0.2% saponin in 1X PBS, and stained as described previously [43,45] using either rabbit anti-myosin 2A (M8064, Sigma-Aldrich, St. Louis, MO) or rabbit anti-pCasL (4015S, Cell Signaling, Danvers, MA) as the primary antibody, and goat anti-rabbit Alexa Fluor 488 (A11034), 555 (A21429) or 594 (A11012) (Molecular Probes, Eugene, OR) as the second antibody. Phalloidin labeled with either Alexa Fluor 488 (A12379) or 568 (A12380) (Molecular Probes, Eugene, OR) was used to stain for F-actin. To inhibit myosin 2, OT1 T cells were preincubated with 25 μM para-nitro-blebbistatin (pnBB) dissolved in DMSO (Optopharma, Budapest, Hungary) or DMSO (Sigma-Aldrich, St. Louis, MO) as the control. 3D-SIM imaging of fixed cells was performed on a DeltaVision OMX 3D-SIM Imaging System (Applied Precision, Issaquah, WA) equipped with an Olympus 60X 1.42 NA objective. Raw data was reconstructed using Softworx (Applied Precision, Issaquah, WA) and using 0.003 for the wiener filter constant. Live cell imaging using TIRF-SIM was performed on either an experimental microscope at the Janelia Research Campus/HHMI [49,51] or a DeltaVision OMX SR TIRF-SIM Imaging System (Applied Precision, Issaquah, WA).

### Image analyses

To qualitatively assess actin arc morphology, max projections of 3D-SIM images of phalloidin-stained OT1 T cells were scored manually into three categories: “Organized”, “Disorganized”, and “No Arcs”. The FibrilTool plugin for ImageJ was used to measure actin arc morphology as described previously [52]. Briefly, the pSMAC regions in max projections of phalloidin-stained 3D-SIM images were divided into 7–8 trapezoid shaped ROIs of similar size to measure the anisotropy of arcs in the radial symmetric pSMAC. To measure the distribution of TCR MCs, whose position was reported indirectly using fluorescent SA to anchor the biotinylated pMHC monomers to the glass-supported lipid-bilayer, we used the radial plot profile plugin for ImageJ. The outline of the T cell was identified manually from F-actin channel, then superimposed on the fluorescent SA channel to determine the radial plot profile. To account for cell size variance, cell radii were normalized to 1. The radial plot profile integral intensities were background subtracted and normalized based on the maximum value. To present the data, the normalized integral intensities were binned (100-bins) along the normalized radii. Unless indicated otherwise, the Student’s Test was used to test for statistical significance between control and experimental conditions. For data sets exhibiting significant differences in variance, an unequal variance T-test known as Welch's T-test was used (as indicated in figure legends). Statistical analyses were performed using Prism 6 (GraphPad, La Jolla, CA).

### Imaging flow cytometry assay

Imaging flow cytometry assay was performed essentially as described previously [45,53]. In brief, OT1 T cells and EL4 cells were transfected overnight with GFP-F-Tractin and RFP-farnesyl plasmids, respectively, using an AMAXA nucleofector kit (Lonza, Walkersville, MD). EL4 cells were pulsed with 2 μg/ml peptide for 1 hour at 37 °C and washed 3 times in medium. OT1 T cells were mixed with peptide-pulsed EL4 cells at a ratio of 1:1 and incubated with NucBlue Live Ready Probes Reagent (R37605, Molecular Probes, Eugene, OR) for 10 min at 37 °C. For myosin 2 inhibition, OT1 T cells were preincubated for 30 min with either 25 μM pnBB or an equivalent volume of DMSO as the control. Samples were fixed with 4% formaldehyde, permeabilized using 0.2% saponin, and stained individually with the following polyclonal antibodies to phosphorylated signaling molecules: anti-phosphorylated Src (Y416) (2101S), anti-phosphorylated Zap70 (Y319)/Syk (Y352) (2701S), and anti-phosphorylated LAT (Y191) (3584S) (Cell Signaling, Danvers, MA). An Alexa Fluor 647-labeled mouse anti-rabbit secondary antibody (A21245, ThermoFisher Scientific, Grand Island, NY) was used. Data was acquired using an Amnis Imagestream Gen-X Mark II flow cytometer (EMD Millipore, Darmstadt, Germany) equipped with 4 lasers (405, 488, 561, and 642 nm). All acquisition was performed using 40X magnification, and by collecting a minimum of 30,000 events with negative control (peptide-null) and compensation controls. Focused cells on the basis of the ‘gradient RMS’ feature were analyzed. Doublet events were gated from the aspect ratio analysis, then refined further using GFP and RFP double-positive cells. OT1-EL4 cell doublets were successfully identified using this strategy, and the selection of good quality, focused doublets within the viewing window allowed the refinement of the final gating (N = 13–294). A combination mask was then used to identify pixels at the interface (valley mask) between the two cells, and at the overlap with the T cell (intensity mask), corresponding to the IS. Background corrected mean fluorescence intensities (MFIs) at the masked region for each of the phosphorylated signaling molecules were calculated by subtracting the MFI of the peptide-null control from the MFIs for each antibody staining. These values were then used for statistical analyses (Prism 6, GraphPad).

### Adhesion conjugation assay

Adhesion conjugate assays were performed essentially as described previously [54,55]. Briefly, EL4 cells were pulsed with 2 μg/ml OVA or G4 peptide for 1 hour at 37 °C, washed three times in medium, mixed with T cells at a ratio of 1:4, and incubated for 30 min at 37 °C. Samples were then fixed with 4% formaldehyde, labeled with 5 μg/ml APC-conjugated anti-mouse H-2K^b^ antibody (AF6-88.5.5.3, eBioscience, San Diego, CA) and 5 μg/ml PE-conjugated anti-mouse CD8 antibody (53–6.7, eBioscience, San Diego, CA) for 30 min, and washed three times in medium. For myosin 2 inhibition, OT1 T cells were preincubated for 30 min with either 25 μM pnBB or DMSO as the control. Conjugate frequency was determined using flow cytometry (LSR II, BD Bioscience, Franklin Lakes, NJ) by collecting at least 30,000 events, with the CD8/H-2K^b^ double-positive population representing OT1: EL4 cell conjugates. Of note, results obtained using unfixed conjugates yielded ~15% lower mean conjugation frequencies, albeit with identical statistical relationships between the four samples measured. The paired Student’s T-test was used to test for statistical significance. Statistical analyses were performed using Prism 6 (GraphPad, La Jolla, CA).

## Results

### Organized actomyosin arcs form in OT1 T cells in response to a strong but not a weak ligand

To address the relationship between TCR–pMHC interaction and the remodeling of actomyosin structures at the IS, we used the well-studied transgenic OT1 CD8+ T cell system with two different ligands for stimulation: chicken ovalbumin peptide fragment OVA^257-264^ (SIINFEKL) as the strong ligand, and the GΔN mutant version of OVA^257-264^ known as G4 (SIIGFEKL) as the weak ligand. Although the differences between these two ligands in terms of structure [56] and TCR interaction kinetics [57–59] are minimal, they differ dramatically in their ability to drive T cell effector function, with OVA^257-264^ being much more potent at inducing IL-2 production [59], upregulating CD69 surface expression and Jun kinase activity [58], increasing CD8 cell surface expression [60], and polarizing the centrosome and lytic granules to the IS [61]. In addition, the binding of the OT1 TCR to OVA:H-2K^b^ induces a catch-bond, whereas the binding to G4:H-2K^b^ induces a slip-bond [24]. Notably, the cell’s ability to apply force on these TCR–pMHC interactions requires the Rho family GTPase Cdc42, an inducer of actin filament assembly [26].

**Fig 1:**
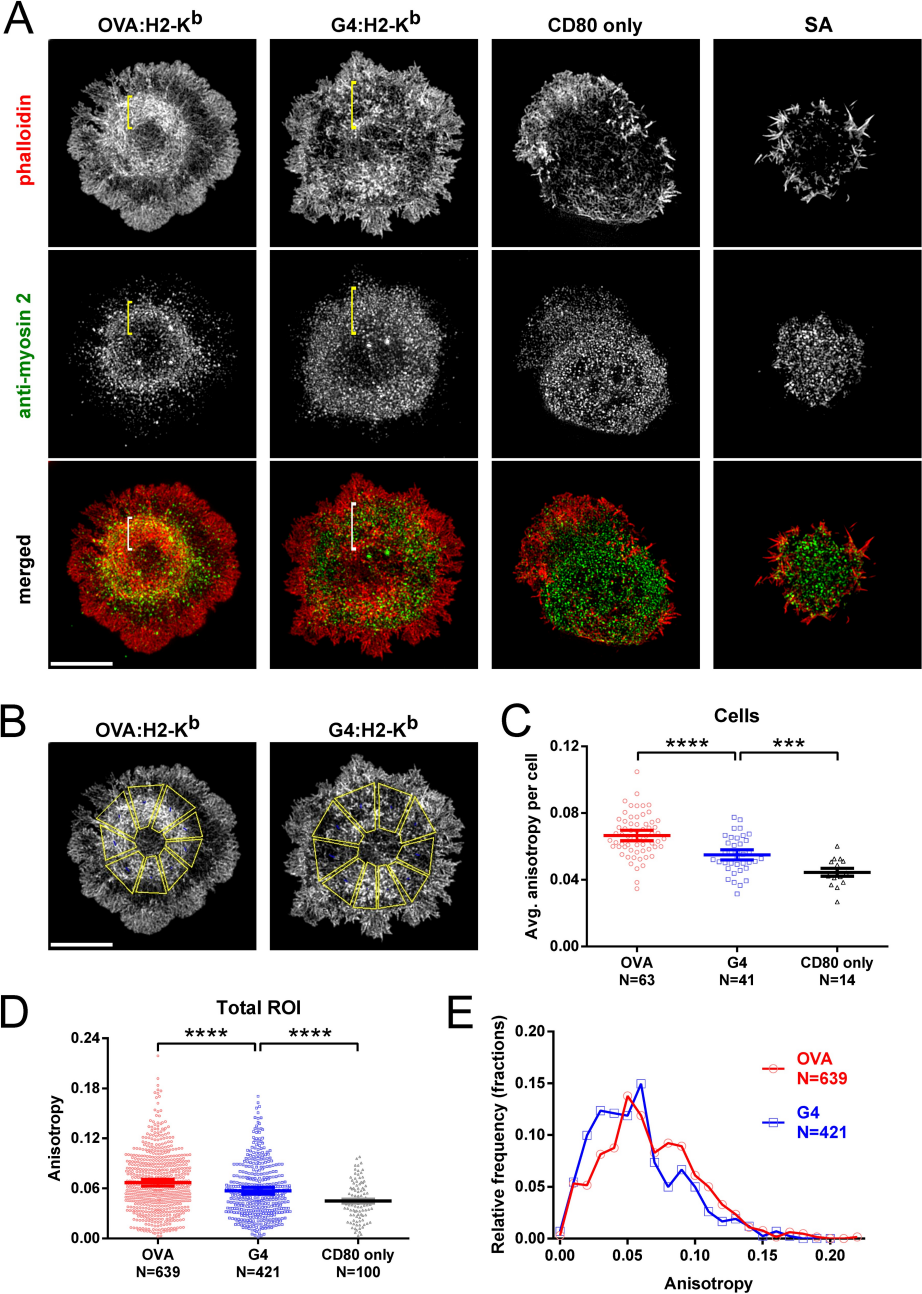
Strong but not weak ligand induces organized, radially-symmetric actomyosin arcs. (A) Representative 3D-SIM images of OT1 T cells fixed 7 min after surface attachment and stained with phalloidin (red) and anti-myosin 2A antibody (green). The merged images are shown in the bottom row. The cells were activated on glass surfaces coated with either OVA:H-2K^b^ plus CD80, G4:H-2K^b^ plus CD80, CD80 only, or streptavidin (SA) only. The pSMAC regions are labeled with yellow or white brackets. Scale bar, 5 μm. (B) Examples for FibrilTool analysis. 7–8 similar-sized trapezoidal ROIs covering the pSMAC portion of the IS were drawn to measure the anisotropy of actin arcs. (C) Average anisotropies of actin arcs in the pSMAC region of OT1 T cells activated on surfaces coated with OVA, G4, or CD80-only. (D) Total anisotropies of actin arcs from all ROIs measured in OT1 T cells activated on surfaces coated with OVA, G4, and CD80-only. (E) Histogram of total anisotropies from all ROIs measured on OVA- and G4-coated surfaces (p < 0.00001). Mean ± SEM. An unequal variance T-test (Welch's T-test) was used. ***, **** indicate p < 0.001, 0.0001.

Based on previous work (see [13] for discussion), we hypothesized that ligand strength should dictate the magnitude of force applied to TCR–pMHC interactions in part by dictating the extent of actomyosin arc formation. To test this hypothesis, we allowed OT1 T cells to spread on either OVA:H-2K^b^-coated or G4:H-2K^b^-coated glass surfaces, fixed the cells 7 min after T cell attachment, stained for endogenous F-actin and endogenous myosin 2A (the major myosin 2 isoform expressed in primary mouse T cells [62]), and imaged the cells using 3D-SIM. As negative controls, we also allowed cells to attach to surfaces coated with CD80 only or SA only (Fig 1A). Like Jurkat T cells stimulated with anti-CD3 [45], the majority of OT1 T cells stimulated with OVA:H-2K^b^ showed well organized actomyosin arcs in the pSMAC region of their IS (Fig 1A, see the column marked OVA:H-2K^b^; see also S1 Movie). Specifically, radially symmetric, myosin 2A-decorated actin arcs were observed in the pSMAC region of their IS (Fig 1A, see the brackets in the column marked OVA:H-2K^b^). In sharp contrast, most OT1 T cells stimulated with G4:H-2K^b^ showed disorganized actin arcs and a more diffuse distribution of myosin 2A (Fig 1A, see the brackets in the column marked G4:H-2K^b^; see also S2 Movie). To quantitate the apparent difference between OVA:H-2K^b^- and G4:H-2K^b^-stimulated OT1 T cells as regards actin arc formation, we measured the anisotropy of the arcs using FibrilTool [52], which measures how well a structure of interest (here, actin arcs) in a given region of interest (ROI) (here, the pSMAC) is organized in parallel arrays. Values can range from 0 when the orientation of the structures in the ROI is random, to 1 when all the structures are oriented in the same direction. To deal with the fact that the IS is radially symmetric, we divided the pSMAC region in 3D-SIM images of fixed cells into 7–8 trapezoid-shaped ROIs of similar size (Fig 1B; see also [45]). Consistent with the representative images in Fig 1A, FibrilTool analysis showed that the average isotropy per cell (Fig 1C), as well as the total isotropy from all ROIs (Fig 1D; histogram in Fig 1E) was significantly higher for OVA:H-2K^b^-stimulated cells than for G4:H-2K^b^-stimulated cells (although arc formation in G4:H-2K^b^ stimulated cells was still significantly more robust than in CD80 only-stimulated cells; Fig 1C and 1D). Therefore, both qualitative and quantitative analyses support the conclusion that the extent of actomyosin arc formation during early stages of IS formation depends on ligand potency.

### Myosin 2 contractility promotes actomyosin arc formation in OT1 T cells in a ligand-dependent manner

**Fig 2:**
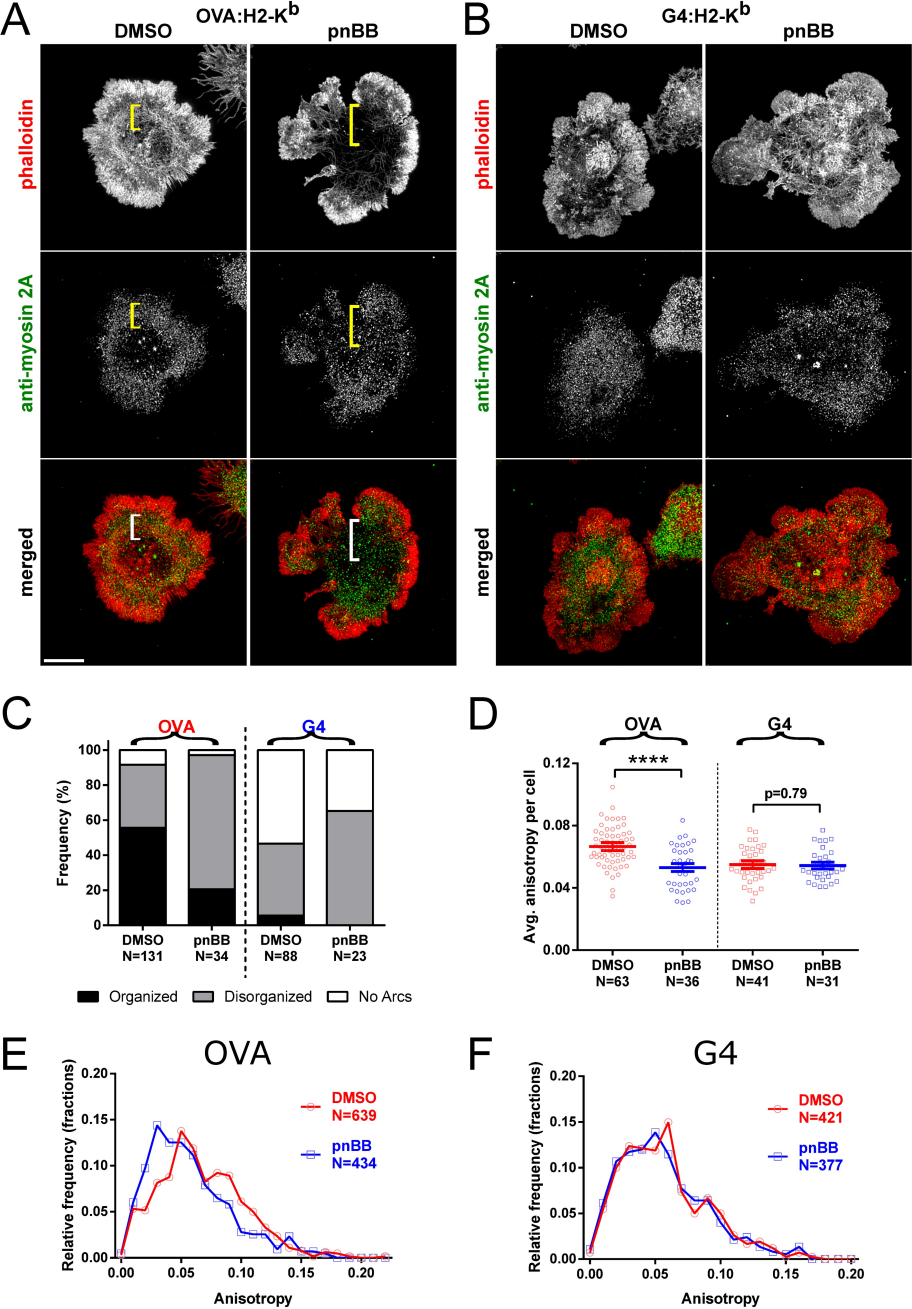
The ATPase activity of myosin 2 promotes actomyosin arc formation in a ligand-dependent manner. (A and B) Representative 3D-SIM images of OT1 T cells that had been pretreated with either DMSO or pnBB in DMSO, allowed to attach to the activating surface for 7 min, and then fixed and stained with phalloidin (red) and anti-myosin 2A antibody (green). The merged images are shown in the bottom row. The cells were activated on glass surfaces coated with either OVA:H-2K^b^ plus CD80 (A) or G4:H-2K^b^ plus CD80 (B). The pSMAC regions are labeled with yellow or white brackets. Scale bar, 5 μm. (C) Frequency of arc morphologies in OT1 cells pretreated with either DMSO or pnBB in DMSO and activated on OVA- or G4-coated surfaces. Arcs were scored as either Organized (i.e. present and concentric), Disorganized (i.e. present but either pointing inwards or entangled), or No arcs (i.e. not present). (D) Average anisotropies of actin arcs in the pSMAC region of OT1 cells pretreated with either DMSO or pnBB in DMSO and activated on OVA- or G4-coated surfaces. (E) Histogram of total anisotropies from all ROIs measured on OVA-coated surfaces with or without pnBB treatment (p < 0.0001). (F) Same as (E) but using G4-coated surfaces (p = 0.44). Mean ± SEM. **** indicates p < 0.0001.

To further define the role played by myosin 2 in ligand-dependent actomyosin arc formation, we used para-nitro-blebbistatin (pnBB), which blocks myosin 2-dependent contractility by locking the myosin in an actin-detached state (note that this new variant of blebbistatin is neither photo-sensitive nor photo-toxic like the original version of blebbistatin [63]). To test whether the effect of pnBB on arc formation depends on ligand potency, OT1 T cells that had been treated with either DMSO or 25 μM pnBB in DMSO for 30 min were allowed to spread for 7 min on either OVA:H-2K^b^-coated or G4:H-2K^b^-coated glass surfaces, and then fixed and stained for F-actin and myosin 2A and imaged using 3D-SIM. In the case of OVA:H-2K^b^-stimulated cells (Fig 2A), pnBB treatment caused a significant disruption in the formation of actomyosin arcs as compared to the control, DMSO-treated cells (Fig 2A, see the brackets in the columns marked “DMSO” and “pnBB”). This apparent difference was supported by qualitative scoring of actomyosin arc organization (Fig 2C, “OVA”) and by quantitative anisotropy measurements (Fig 2D, “OVA”; Fig 2E). In contrast to these results, the already reduced extent of actomyosin arc formation exhibited by G4:H-2K^b^-stimulated OT1 T cells (see Fig 1) appeared to be minimally affected by pnBB treatment (Fig 2B, see the brackets in the columns marked “DMSO” and “pnBB”). This conclusion was borne out by qualitative scoring of actomyosin arc organization (Fig 2C, “G4”) and by anisotropy measurements (Fig 2D, “G4”; Fig 2F), which showed that the extent of actin arc formation exhibited by G4:H-2K^b^-stimulated cells was not significantly affected by pnBB treatment. Together, these results argue that myosin 2-dependent contractility promotes actin arc formation in the pSMAC region of OT1 T cells in a ligand-dependent manner.

### Actomyosin arcs in OT1 T cells generate tension at the IS in a ligand-dependent manner

The protein lymphocyte-specific Crk-associated substrate (CasL) can been used as an indirect measure of force generation at the IS because a conformational change in the molecule induced by tension exposes a phosphorylation site whose subsequent modification can be measured quantitatively using an anti-phospho-CasL antibody [64]. For example, Yu and colleagues showed that phosphorylated CasL colocalizes with TCR MCs, and that BB treatment reduces the content of pCasL at the IS [43]. More recently, Santos and colleagues showed that CasL is phosphorylated at TCR MCs in an actin polymerization-dependent manner, and that this event may participate in a positive feedback loop leading to an amplification of Ca^2+^ signaling, inside-out integrin activation, and actomyosin contraction [41]. Whether differences in ligand potency that result in differences in actomyosin arc formation also result in differences in the amount of force generated at the IS has not been determined, however.

**Fig 3:**
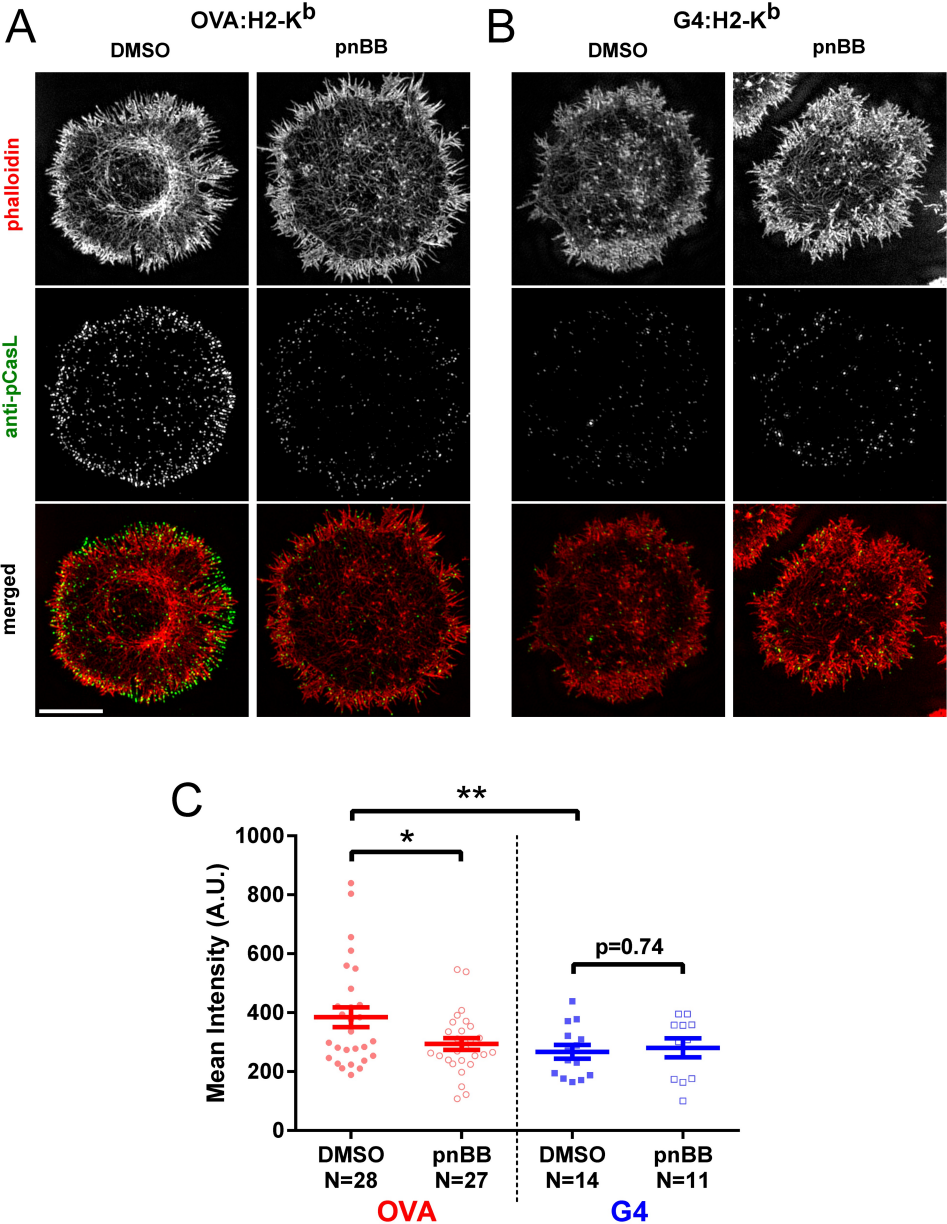
Actomyosin arcs promote tension at the IS in a ligand-dependent manner. (A and B) Representative 3D-SIM images of OT1 T cells that had been pretreated with either DMSO or pnBB in DMSO, allowed to attach to the activating surface for 7 min, and then fixed and stained with phalloidin (red) and anti-pCasL antibody (green). The merged images are shown in the bottom row. The cells were activated on glass surfaces coated with either OVA:H-2K^b^ plus CD80 (A) or G4:H-2K^b^ plus CD80 (B). Scale bar, 5 μm. (C) Mean intensities of pCasL at the IS from 3D-SIM images of OT1 cells pretreated with either DMSO or pnBB in DMSO and activated on OVA- or G4-coated surfaces. Mean ± SEM. An unequal variance T-test (Welch's T-test) was used. * and ** indicate p < 0.05 and < 0.01, respectively.

To correlate ligand-dependent actomyosin arc formation with force generation at the IS, OT1 T cells that had been treated with either DMSO or 25 μM pnBB for 30 min were allowed to spread for 7 min on either OVA:H-2K^b^-coated or G4:H-2K^b^-coated glass surfaces, and then fixed and stained for F-actin and phosphorylated CasL and imaged using 3D-SIM. Consistent with the effect of ligand potency on the formation of actomyosin arcs seen in previous figures, representative OVA:H-2K^b^-stimulated cells (Fig 3A, “DMSO”) showed significantly stronger staining for pCasL than representative G4:H-2K^b^-stimulated cells (Fig 3B, “DMSO”). Moreover, pCasL levels appeared to decrease more dramatically in OVA:H-2K -stimulated cells treated with pnBB (Fig 3A, “pnBB”) than in G4:H-2K^b^-stimulated cells treated with pnBB (Fig 3B, “pnBB”). These conclusions were both borne out by quantitation of the amount of pCasL staining at the IS (Fig 3C), which revealed a significantly lower level in control G4:H-2K^b^-stimulated cells than in control OVA:H-2K^b^-stimulated cells, as well as a significantly lower level in pnBB-treated OVA:H-2K^b^-stimulated cells than in control OVA:H-2K^b^-stimulated cells, but no significant difference between control G4:H-2K^b^-stimulated cells and pnBB-treated G4:H-2K^b^-stimulated cells. Together, these results argue that actomyosin arcs in OT1 T cells generate tension at the IS in a ligand-dependent manner.

### Actomyosin arcs facilitate the accumulation of ligands at the IS center in a ligand-dependent and contractility-dependent manner

**Fig 4:**
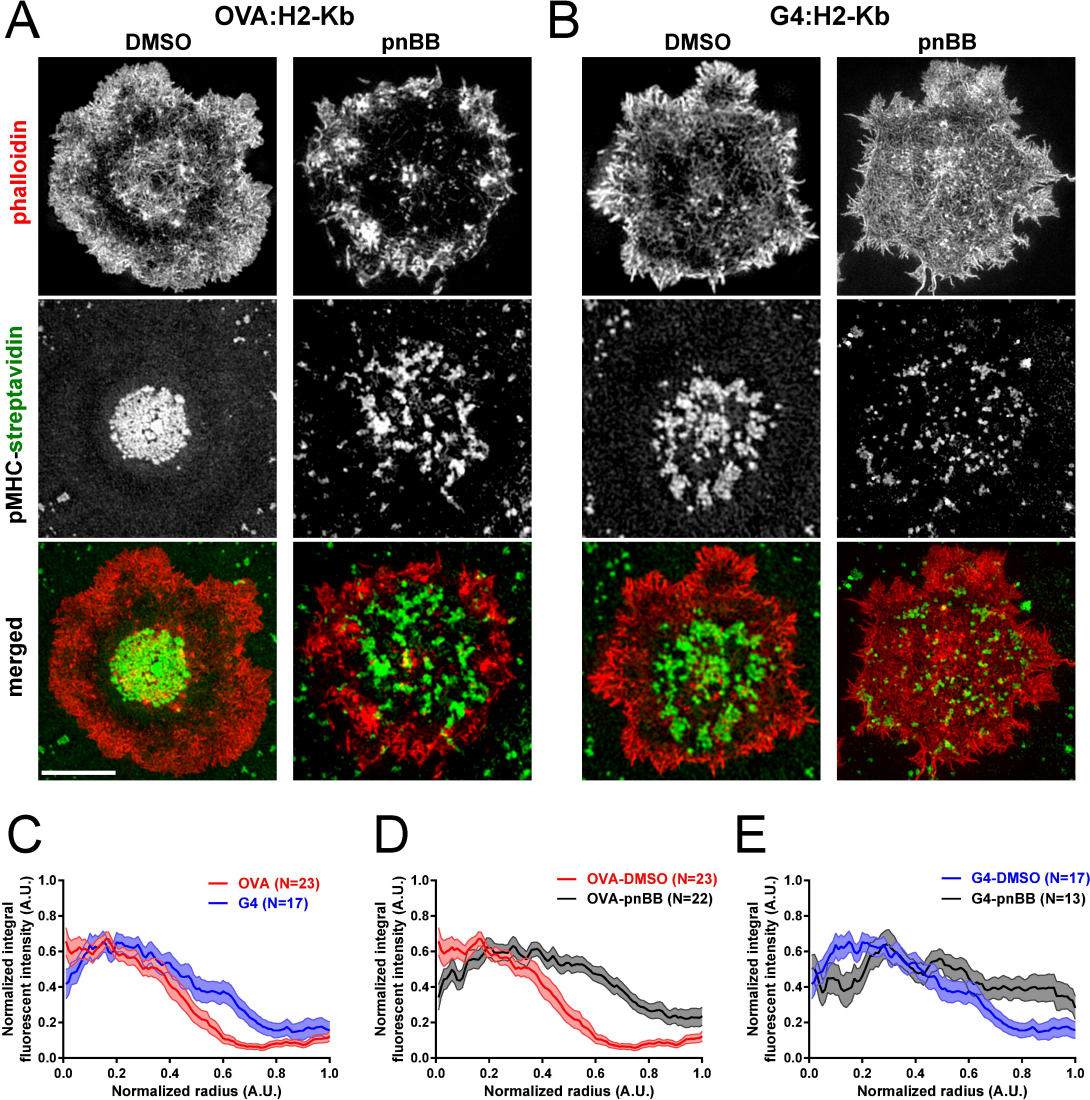
Actomyosin arcs facilitate the accumulation of TCR MCs at the IS center in a ligand-dependent and contractility-dependent manner. (A and B) Representative 3D-SIM images of OT1 T cells that had been pretreated with either DMSO or pnBB in DMSO, allowed to attach for 7 min to planar lipid bilayers containing ICAM-1 and either OVA:H-2K^b^ (A) or G4:H-2K^b^ (B) bound via fluorescent SA (to indirectly report the position of TCR MCs in the T cell), and then fixed and stained with phalloidin (red). The distribution of SA (green) and the merged images are shown in the middle and bottom rows, respectively. Scale bar, 5 μm. (C) Averaged, normalized radial integral intensity profiles of fluorescent SA in bilayers containing OVA:H-2K^b^ (red) or G4:H-2K^b^ (blue) (p <0.001). (D) Averaged, normalized radial integral intensity profiles of fluorescent SA in bilayers containing OVA:H-2K^b^ using OT1 T cells pretreated with either DMSO (red) or pnBB in DMSO (black) (p < 0.0001). (E) Averaged, normalized radial integral intensity profiles of fluorescent SA in bilayers containing G4:H-2K^b^ using OT1 T cells pretreated with either DMSO (blue) or pnBB in DMSO (black) (p < 0.0001). For (C), (D) and (E), the mean value is represented by the bold line, while the SEM is represented by the surrounding shaded area.

To further examine the consequences of differential TCR activation on the function of actomyosin arcs, we quantitated the degree to which TCR MCs accumulate at the center of the IS in OT1 T cells 7 min after engagement with a glass-supported lipid bilayer containing fluorescently-labeled SA bound to either the strong or the weak ligand. OT1 T cells activated with the strong ligand OVA:H-2K^b^ typically exhibited a pronounced accumulation of TCR MCs at the center of the IS, i.e. at the cSMAC (Fig 4A, “DMSO”), while OT1 T cells activated with the weak ligand G4:H-2K^b^ typically exhibited a much looser accumulation of TCR MCs (Fig 4B, “DMSO”). Averaged normalized radial intensity profiles of TCR MC distribution across the IS showed that this difference was statistically significant (Fig 4C, compare “OVA” in red to “G4” in blue; the dark line shows the mean, while the shaded area shows the SEM). Consistent with a subset of previous studies addressing the role of myosin 2 in TCR MC transport [42,44,45], OVA:H-2K^b^-stimulated OT1 cells that were pretreated with pnBB typically exhibited a striking decrease in the central accumulation of TCR MCs relative to the DMSO control (Fig 4A, “pnBB”). This apparent difference was supported by averaged normalized radial intensity profiles (Fig 4D, compare “OVA-DMSO” in red to “OVA-pnBB” in black). Interestingly, G4:H-2K^b^-stimulated OT1 T cells that were pretreated with pnBB also showed a further degradation of inward TCR MC transport relative to the DMSO control (Fig 4B, “pnBB”) that was statistically significant (Fig 4E, compare “G4-DMSO” in blue to “G4-pnBB” in black). Although the amount of tension exerted at the IS is lower on glass-supported lipid bilayers than on glass (S1 Fig), these results still support the idea that ligand-dependent actomyosin arcs and the force they generate facilitate IS maturation by transporting and constraining TCR MCs, most likely in a self-reinforcing manner.

### Myosin 2 facilitates proximal TCR signaling and T cell: APC adhesion in a ligand-dependent manner

**Fig 5:**
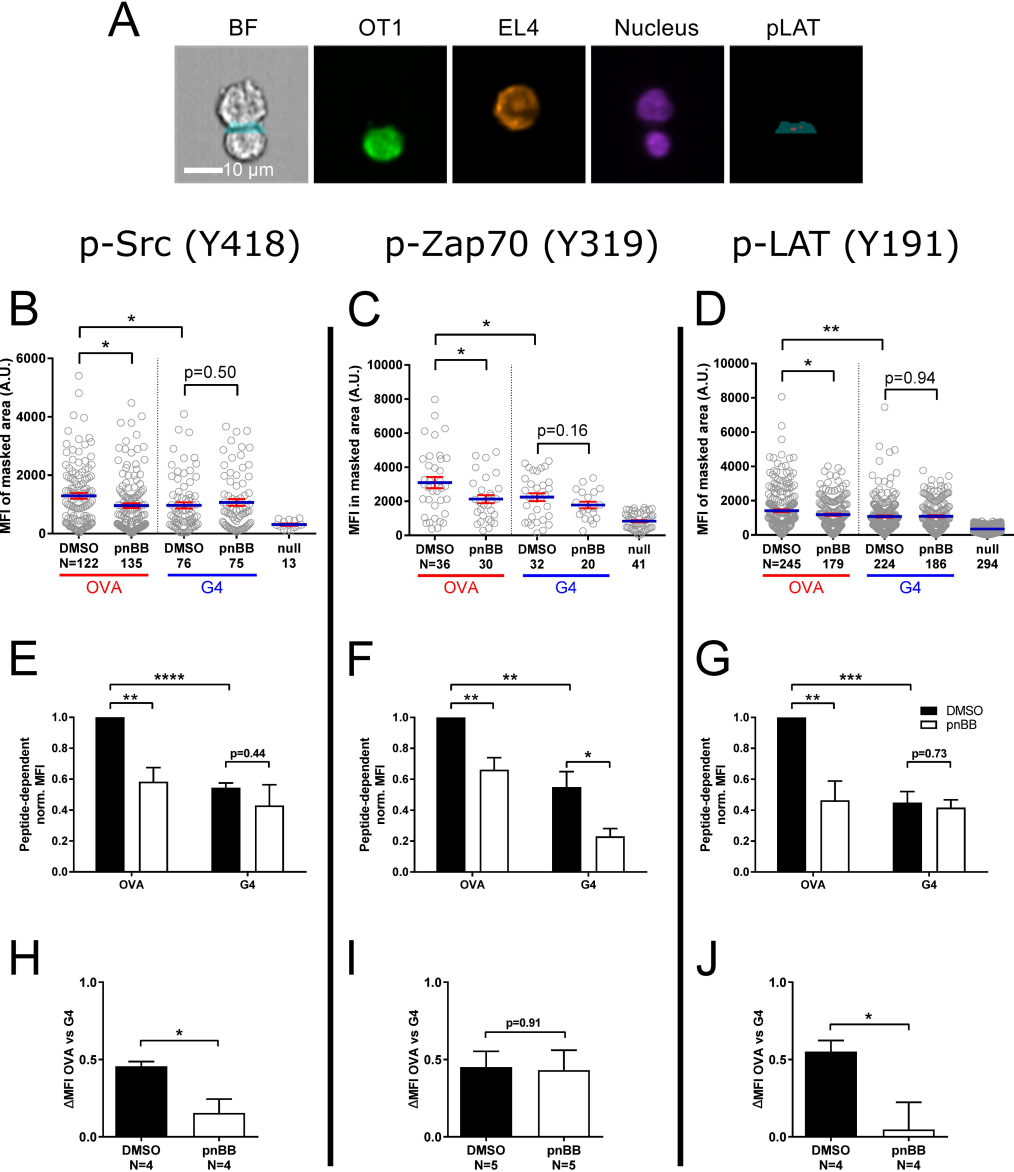
Myosin 2 facilitates proximal TCR signaling in a ligand-dependent manner. (A) Representative example of AMNIS Imagestream images used to quantitate signaling molecule phosphorylation at the IS of OT1 T cells conjugated with EL4 target cells by employing a combination masking strategy (see Methods for details). Shown from left to right are the bright-field (BF) image with the portion of the combination mask that corresponds to the IS (blue), the signal for GFP-F-Tractin (green) in the OT1 T cell, the signal for farnesylated RFP (pseudocolored orange) in the EL4 target cell, the signals for the nucleus (purple) in both cells, and the portion of the combination mask that corresponds to the IS (blue) overlaid with the signal for pLAT at the IS (red). Scale bar, 10 μm. (B, C, and D) MFIs for pSrc (B), pZap70 (C), and pLAT (D) at the IS of OT1 T cells that had been pretreated with either DMSO or pnBB in DMSO and allowed to form conjugates for 10 min with EL4 target cells loaded with either OVA:H-2K^b^ or G4:H-2K^b^ prior to fixation and staining. Each circle represents a single OT1: EL4 conjugate. (E, F, G) Normalized MFIs from three or more experiments performed exactly as in (B), (C) and (D), except that these normalized MFIs were background corrected by subtracting the MFI for the null peptide control. (H, I, and J) Shown are the differences in mean MFI values presented in (H), (I) and (J) between OVA:H-2K^b^-engaged and G4:H-2K^b^-engaged T cells. The value obtained, referred to as “ΔMFI OVA vs G4” on the y axis, represents a measure of the contribution made by myosin 2 contractility to ligand discrimination. Mean ± SEM (N ≥ 3). *, **, *** indicate p < 0.05, 0.01, 0.001.

We next asked if myosin 2 contributes to TCR proximal signaling in a ligand-dependent manner. To accomplish this, OT1 T cells expressing GFP-tagged F-Tractin and pretreated with either DMSO or 25 μM pnBB in DMSO for 30 min were mixed with EL4 target cells expressing the plasma membrane marker farnesylated-RFP and loaded with either OVA:H-2K^b^, G4:H-2K^b^ or null peptide. Following a 10-minute incubation, the cells were fixed and immunostained with fluorescent antibodies specific for the tyrosine phosphorylated versions of Src (Y416), Zap70 (Y319), or Lat (Y191) as proxies for the extent of proximal TCR signaling [65,66]. To quantitate the signals for pSrc, pZap70 and pLat at the IS, we used an Amnis Imagestream flow cytometer, which combines the sample size advantages of flow cytometry with the sensitivity of fluorescence microscopy [53]. Specifically, we identified GFP- and RFP-double positive OT1: EL4 cell conjugates and measured the mean fluorescence intensity (MFI) of the three phosphorylated signaling molecules at the IS by employing a masking strategy to identify that portion of the contact area between the two cells that corresponds to the T cell’s IS (Fig 5A) (see [45] and Methods for further details). Fig 5B, 5C and 5D show the results of a typical experiment for pSrc, pZap 70 and pLat, respectively (each point represents a single, double-positive conjugate). As expected, the MFI for all three proteins was significantly lower at the IS of DMSO-treated OT1 cells engaged with G4:H-2K^b^-loaded target cells than at the IS of DMSO-treated OT1 cells engaged with OVA:H-2K^b^-loaded target cells. Moreover, consistent with previous studies in Jurkat T cells [42], the MFI for all three proteins was significantly lower at the IS of OVA:H-2K^b^-engaged OT1 cells that had been pretreated with pnBB for 30 min than at the IS of OVA:H-2K^b^-engaged OT1 that had been pretreated with DMSO. In contrast, the MFI for all three proteins was not significantly lower at the IS of G4:H-2K^b^-engaged OT1 cells that had been pretreated with pnBB than at the IS of G4:H-2K-engaged OT1 that had been pretreated with DMSO. With one exception, these results were also observed in normalized data pooled from three or more independent experiments (Fig 5E, 5F and 5G; note that the null peptide signal was subtracted in calculating this pooled data). The one exception was with pZAP70, which ended up being significantly lower at the IS of G4:H-2K^b^-engaged OT1 cells that had been pretreated with pnBB than at the IS of G4:H-2K-engaged OT1 that had been pretreated with DMSO (Fig 5F). Together, these results indicate that the magnitude of myosin 2’s contribution to proximal TCR signaling depends significantly on ligand potency for two early signaling molecules, Src and LAT.

To further illuminate the ligand-dependency of myosin 2’s contribution to proximal signaling, we used the normalized data in Fig 5E–5G to calculate the difference in MFI between OVA:H-2K^b^-engaged T cells and G4:H-2K^b^-engaged T cells for both DMSO- and pnBB-treatment conditions (Fig 5H–5J). The value obtained, which we refer to as “ΔMFI OVA vs G4”, is in essence a measure of the contribution made by myosin 2 contractility to ligand discrimination- the bigger the discrepancy in this value between pnBB-treated cells and control, DMSO-treated cells, the greater is myosin 2’s contribution to ligand discrimination. Consistent with the idea that myosin 2 contractility helps T cells distinguish between ligands of different potency, pnBB-treated T cells exhibited a ΔMFI OVA vs G4 value for pSrc that was ~three times smaller than the value for control, DMSO-treated cells (Fig 5H). For pLAT, pnBB-treated T cells exhibited a ΔMFI OVA vs G4 value that was almost ten-times smaller than the value for control, DMSO-treated cells (Fig 5J). Only pZAP70 did not follow this trend (Fig 5I). Of note, pCasL followed the same trend, with pnBB-treated T cells exhibiting a ΔMFI OVA vs G4 value that was almost five-times smaller than the value for control, DMSO-treated cells (S2 Fig). In summary, the extent to which myosin 2 promotes the activation of two key early signaling molecules depends on ligand potency, with the stronger ligand being much more dependent on myosin 2. In other words, the ability of the cell to distinguish between OVA:H-2K^b^ and G4:H-2K^b^, as evidenced by the phosphorylation of Src and LAT, is facilitated significantly by myosin 2 contractility. As for why pZAP70 differs from pLat and pSrc, one possibility is that the activation of ZAP70 is more sensitive than Lat and Src to mechanical strain generated at the IS by myosin contractility and actin arc flow. As a result, the relatively modest difference in strain at the IS that probably exists between pnBB- and DMSO-treated T cells engaged with the weak G4 ligand is sufficient to see a difference in the activation of ZAP70, but not in the activation of Lat or Src.

**Fig 6:**
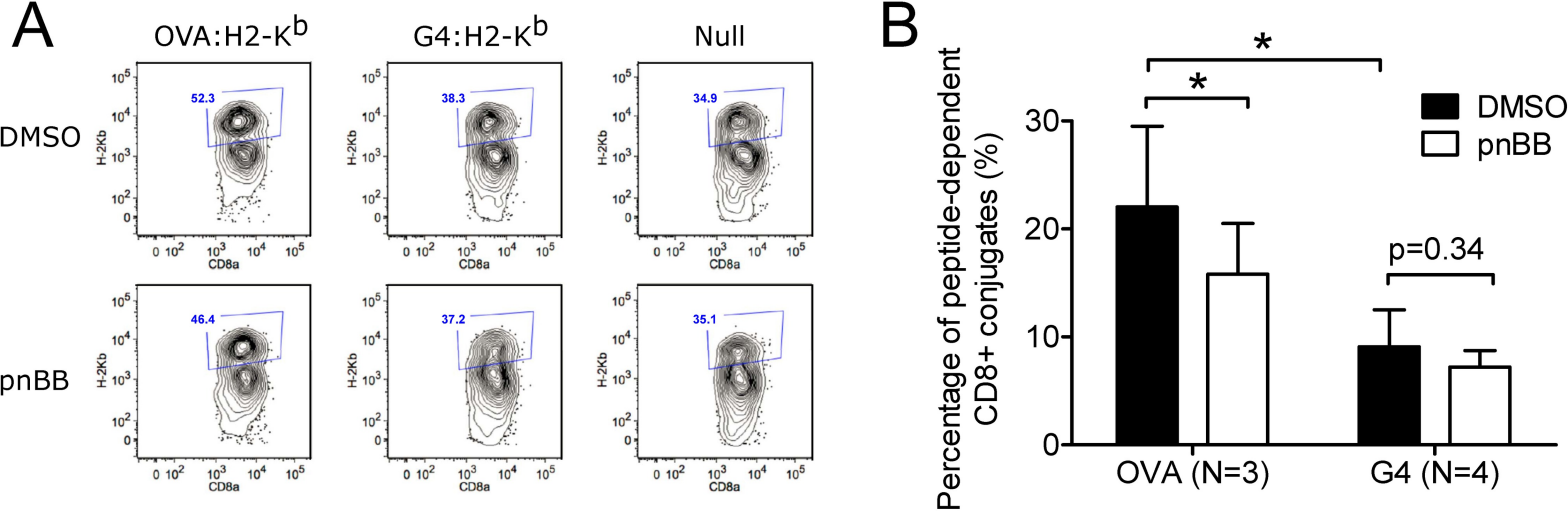
Myosin 2 facilitates T cell adhesion in a ligand dependent manner. (A) Shown are scatter plot profiles from conjugation assays obtained using flow cytometry (see Methods for details). OT1 T cells pretreated with either DMSO or pnBB in DMSO were incubated for 30 min with EL4 target cells loaded with either OVA peptide, G4 peptide, or null peptide. The cell population in the anti-CD8/H-2K^b^ double-positive staining channel (gated as blue square) corresponds to OT1: EL4 conjugates. (B) Conjugation frequency (note that these values were background corrected by subtracting the percent conjugation obtained using the null peptide control). Mean ± SEM (N ≥ 3). * indicates p < 0.05. We note that steric hindrance from the bulky antibodies employed in this assay could result in a reduction in conjugation frequency.

Lastly, to test the contribution made by myosin 2 to T cell: APC adhesion, and the extent to which this contribution depends on ligand potency, we performed *in vitro* conjugation assays using flow cytometry. To accomplish this, OT1 T cells pretreated with either DMSO or pnBB for 30 min were mixed with EL4 target cells loaded with either OVA:H-2K^b^, G4:H-2K^b^ or null peptide, and the mixture incubated for 30 min to allow conjugate formation. Following fixation and labeling with fluorescent anti-CD8a and anti-H-2K^b^ antibody, flow cytometry was used to determine the percentage of total CD8^+^ OT1 T cells that were in anti-CD8a/anti-H-2K^b^ double-positive conjugates (Fig 6A). As expected, the percentage of OT1 T cells in conjugates was significantly lower for G4:H-2K^b^-loaded target cells than for OVA:H-2K^b^-loaded target cells (Fig 6B; these values were background corrected by subtracting the value obtained with the null peptide). Moreover, consistent with previous studies in Jurkat T cells [42], the percentage of OT1 T cells in conjugates was significantly lower for OVA:H-2K^b^-engaged OT1 cells that had been pretreated with pnBB for 30 min than for OVA:H-2K^b^-engaged OT1 that had been pretreated with DMSO. In contrast, the percentage of OT1 T cells that formed conjugates was not significantly lower for G4:H-2K^b^-engaged OT1 cells that had been pretreated with pnBB than for G4:H-2K-engaged OT1 that had been pretreated with DMSO (although the trend was towards reduced conjugate formation following pnBB treatment) (Fig 6B). We conclude, therefore, that myosin 2 contributes to T cell: APC adhesion in a ligand-dependent manner. As for the significant degree of adhesion exhibited by pnBB-treated, OVA:H-2K^b^-engaged OT1 T cells, we speculate that the polymerization-driven actin retrograde flow that persists in myosin 2-inhibited cells [29,38] is sufficient over the 30 min time course of this experiment to activate many integrins [67].

## Discussion

Recent studies in Jurkat T cells [29,38,45] have revealed the presence of actomyosin 2 arcs in the pSMAC region of the IS that, together with actin polymerization-driven retrograde flow in the dSMAC, drive the centralization of TCR MCs and integrin clusters during IS maturation. Here we sought to define the contribution made by these actomyosin 2 arcs to the function of the IS in the context of primary mouse T cells, which we recently showed also contain actomyosin arcs in their pSMAC [45]. More specifically, we sought to define the ligand-dependent contribution made by these contractile structures to TCR MC transport and proximal signaling by activating primary mouse OT1-CD8^+^ T cells with two OVA peptides exhibiting different affinities for the TCR. By combining this approach with super-resolution microscopy and the use of pnBB to conditionally block myosin 2-dependent force generation, we showed that the formation of organized actomyosin arcs depends on ligand potency and the ability of myosin 2 to contract actin filaments. While weak ligands induced disorganized actomyosin arcs, strong ligands resulted in organized actomyosin arcs that correlated well with tension-sensitive CasL phosphorylation and the accumulation of TCR MCs at the IS center. Finally, we showed that blocking myosin 2 contractility greatly reduced the difference in the extent of Src and LAT phosphorylation observed between the strong and the weak ligand. This key observation argues that myosin 2-dependent force generation within actin arcs at the pSMAC contributes to ligand discrimination. Together, our results underscore the growing consensus [11,13,47] that actomyosin contractility at the IS contributes to differential TCR activation and ligand discrimination.

Single-molecule studies have demonstrated that ligand potency-dependent forces regulate the nature and lifetime of the TCR–pMHC bond, with strong ligands creating long-lived catch bonds and weak ligands creating short-lived slip bonds [17,24,25,68]. With these studies in mind, it seems reasonable to suggest that the actomyosin arcs described here generate forces in the plane of the IS that either increase or decrease TCR–pMHC bond lifetimes depending on ligand potency. Consistent with this idea, high-resolution TIRF-SIM movies of bilayer-engaged Jurkat T cells show that TCR MCs are largely constrained to the space between the arcs and are swept inward by the arcs towards the IS center [45]. We speculate that this process creates strain on the TCR–pMHC bond that could serve to test the strength of their interaction, i.e. to promote ligand discrimination. In the case of high affinity ligands, the strain generated by actomyosin arcs would drive the formation of long-lived catch bonds, thereby promoting sustained TCR-dependent signaling [37]. In the case of weak ligands, this strain would promote the formation of short-lived slip bonds, leading to the rapid cessation of TCR-dependent signaling. This mechanism could also serve to control the balance between TCR signaling and degradation at the IS center, which is determined by antigen quality [69], and the cluster-dependent phosphorylation of CD3ζ [70]. Future efforts must seek to extend the correlations we present here by creating experiments in which the effect of ligand potency on the formation of actomyosin arcs is somehow uncoupled from its effect on TCR–pMHC interaction. Moreover, interventions other that pnBB that could inhibit myosin 2 contractility without altering the concentric organization of the actomyosin arcs would provide more definitive results. Perhaps most ideal would be a merger of super-resolution imaging, a DNA based-force sensor [26], and ultra-fast single-molecule lifetime imaging [71], as this might allow one to show that regions where actomyosin arcs appear to generate strain on TCR MCs [45] exhibit a lengthening of TCR–OVA:H-2K^b^-pMHC bond lifetime, and a shortening of TCR–G4:H-2K^b^-pMHC bond lifetime. Combining such an approach with the introduction of physical barriers in the bilayer to increase locally the arc-dependent strain on TCR–pMHC bonds [34,40] could help cement the cause-effect relationship between actomyosin force generation and ligand discrimination.

Although functional connections between the actin cytoskeleton and TCR signaling/dynamics have been revealed by studies of TCR–pMHC kinetics [68,72], T cell signaling [37,73], TCR mechano-transduction [15,18], and TCR MC transport [29,38,40,42,43,45,74], the nature of their physical interaction remains largely unknown [75,76]. Future studies should seek to define the molecules that drive this physical interaction, as this information will help elucidate how the TCR translates mechanical information into chemical information. Finally, we note that our data implicating actomyosin arcs in promoting T cell activation in a ligand dependent manner in no way excludes important contributions from other cytoskeletal proteins in this process. For example, differences in intracellular calcium flux that occur downstream of strong versus weak ligand engagement [24,77–79] could lead to differences in the activation of calcium-sensitive F-actin severing proteins like cofilin and the F-actin stabilizing protein TAGLN [80] that probably also serve to promote ligand-dependent T cell activation.

## Supporting information

S1 Fig T cells activated with ligands attached to lipid bilayers induce a lower level of pCasL. Normalized pCasL antibody staining for OT1 T cells activated using OVA:H-2K^b^-coated glass or and OVA:H-2K^b^ plus ICAM-1 attached to lipid bilayers. Mean ± SEM. *** indicates p < 0.001. As expected, the tension generated on bilayers (as inferred from pCasL), where ligands are free to move, is significantly lower than the tension generated on glass, where the ligands are immobilized.
(TIF)

S2 Fig Myosin 2 inhibition reduces the difference in the pCasL level observed between strong and weak ligand activation. Shown is the difference in mean pCasL level between OVA:H-2K^b^-engaged and G4:H-2K^b^-engaged T cells that had been pretreated with either DMSO or pnBB in DMSO. * indicates p < 0.05. This result shows that myosin 2-based contractility influences tension generation at the IS in a ligand-dependent manner.
(TIF)

S1 Movie The strong ligand induces organized, radially-symmetric actomyosin arcs. Shown is a TIRF-SIM movie (20 frames per min, 3 min duration) of an OT1 T cell expressing the dynamic F-actin reporter GFP-F-Tractin (pseudocolored red) and Halo-tagged myosin 2A (pseudocolored green) and activated on a glass surface coated with OVA:H-2K^b^ and CD80. The Halo dye 567 was added to the media 30 min before imaging to visualize Halo-tagged myosin 2A. Consistent with the still images in Fig 1, the strong ligand induced the formation of dynamic, well-organized, radially-symmetric pSMAC actin arcs that are highly decorated with myosin 2.
(AVI)

S2 Movie The weak ligand induces disorganized actin arcs and more diffuse myosin 2 distribution. Shown is a TIRF-SIM movie (15 frames per min, 3 min duration) of an OT1 T cell expressing the dynamic F-actin reporter GFP-F-Tractin (pseudocolored red) and Halo-tagged myosin 2A (pseudocolored green) and activated on a glass surface coated with G4:H-2K^b^ and CD80. The Halo dye 567 was added to the media 30 min before imaging to visualize Halo-tagged myosin 2A. Consistent with the still images in Fig 1, the weak ligand induced the formation of poorly-organized pSMAC actin arcs and a shift in myosin 2 localization towards a more diffuse pattern.
(AVI)
