# Supplementary figures and images for: Contractile actomyosin arcs promote the activation of primary mouse T cells in a ligand-dependent manner
Source: PLoS One. 2017 Aug 17;12(8):e0183174. doi: 10.1371/journal.pone.0183174 (PMC5560663; doi:10.1371/journal.pone.0183174)

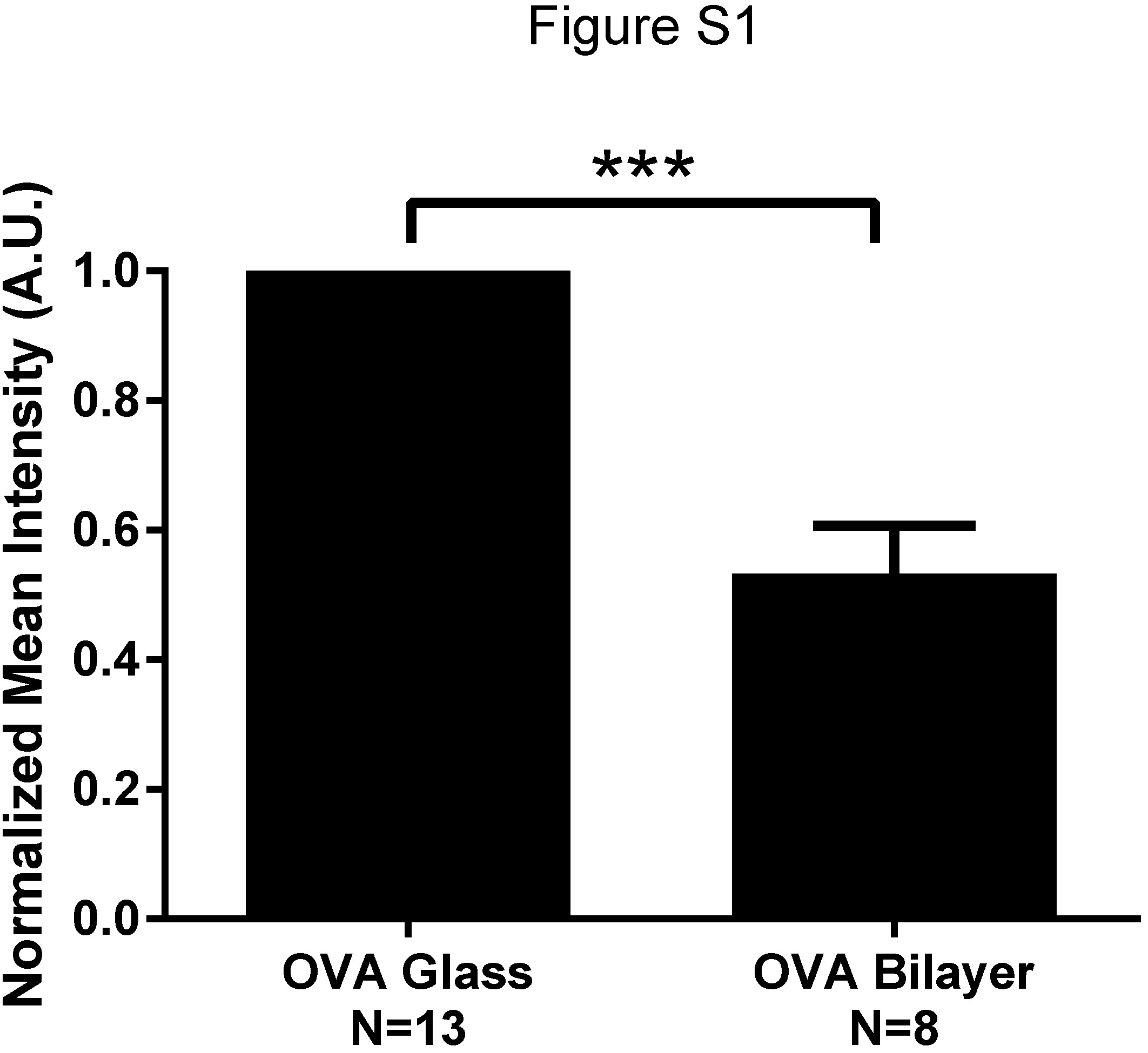

Supplement: S1 Fig — Normalized pCasL antibody staining for OT1 T cells activated using OVA:H-2Kb-coated glass or and OVA:H-2Kb plus ICAM-1 attached to lipid bilayers. Mean ± SEM. *** indicates p < 0.001. As expected, the tension generated on bilayers (as inferred from pCasL), where ligands are free to move, is significantly lower than the tension generated on glass, where the ligands are immobilized. (TIF) [file pone.0183174.s001.tif]

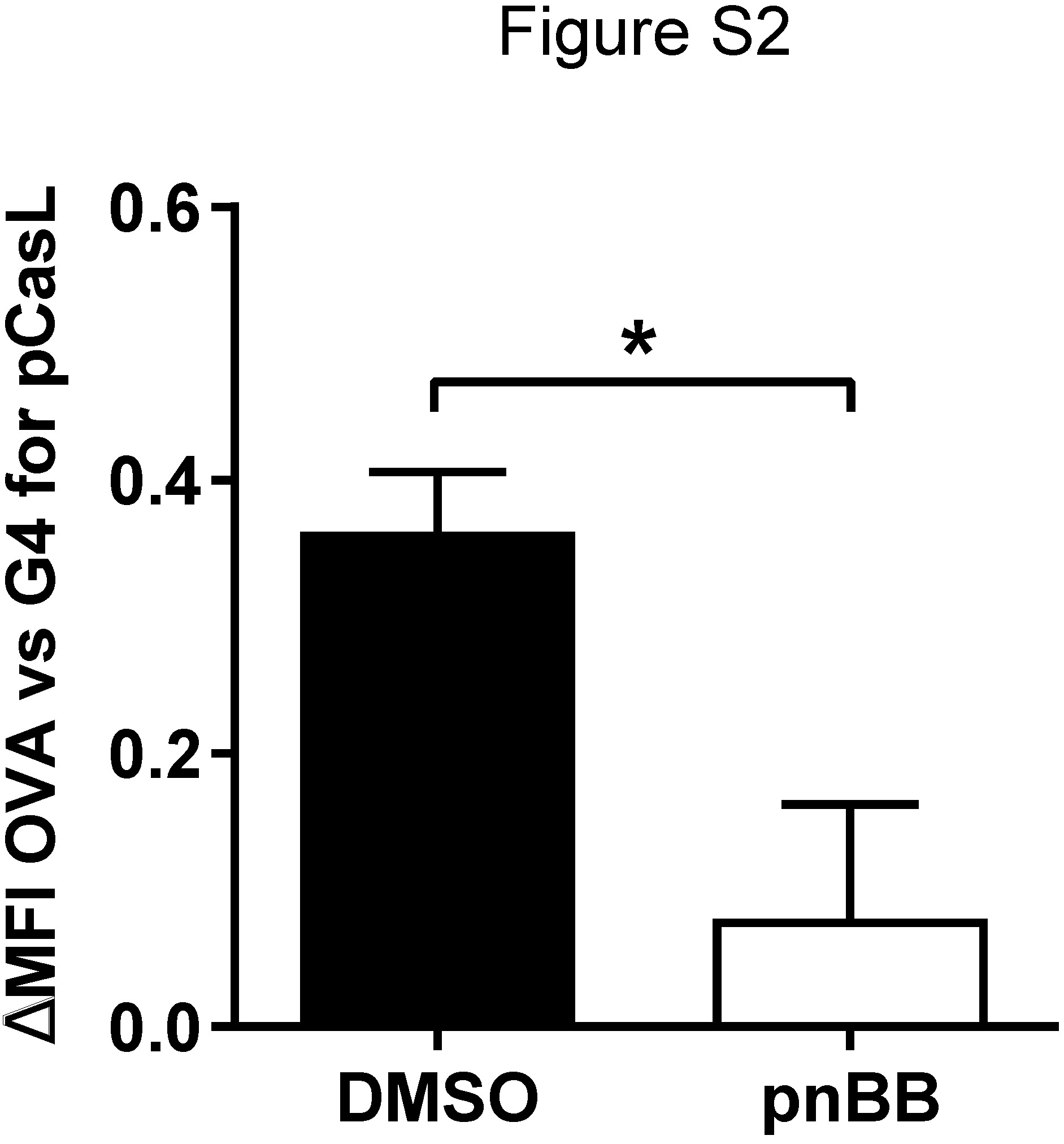

Supplement: S2 Fig — Shown is the difference in mean pCasL level between OVA:H-2Kb-engaged and G4:H-2Kb-engaged T cells that had been pretreated with either DMSO or pnBB in DMSO. * indicates p < 0.05. This result shows that myosin 2-based contractility influences tension generation at the IS in a ligand-dependent manner. (TIF) [file pone.0183174.s002.tif]
